# Vitamin D_3_ supplementation improves spatial memory, muscle function, pain score, and modulates different functional physiological biomarkers in vitamin D_3_ deficiency diet (VDD)-induced rats model

**DOI:** 10.1186/s40795-023-00767-0

**Published:** 2023-09-25

**Authors:** Mahendra Kumar Trivedi, Alice Branton, Dahryn Trivedi, Sambhu Mondal, Snehasis Jana

**Affiliations:** 1 Trivedi Global, Inc, Henderson, NV USA; 2 Trivedi Science Research Laboratory Pvt. Ltd, Thane (W), Maharashtra India

**Keywords:** Cholecalciferol, Vitamin d_3_ deficiency diet, klotho, VDR, Spatial memory

## Abstract

**Background:**

Vitamin D Deficiency is recognized as a pandemic, which is associated with high mortality. An inadequate level of vitamin D is associated with autoimmune diseases, hypertension, and cancer. The study was aimed to assess the pharmacological effects of chronic vitamin D_3_ supplementation on the manipulation diet regiment of deprived cholecalciferol (vitamin D_3_ deficient diet, VDD) rats.

**Methods:**

Memory performance (Y-maze task), muscular function (muscle grip strength), and pain score (pressure application measurement meter) were measured. Functional biomarkers were measured using ELISA method in different matrix viz. in serum (parathyroid hormone; PTH, calcitonin, thyroxine, and C-reactive protein; CRP, 25-OH Vit D_3_), and in CSF (klotho and β-endorphin). 25-OH Vit D_3_ was also estimated in liver and kidney homogenate using ELISA. Vitamin D receptor (VDR) was measured spectrophotometrically in liver and adipose tissue.

**Results:**

VDD-induced rats showed a decrease in number of entries and time spent in the novel arm and spontaneous alternations in the Y-maze task. Significant improvements of neuromuscular function and pain score after addition of vitamin D_3_. In comparison to the VDD group, VDR expression (liver) and active metabolites of vitamin D_3_ (25-OH vit.D_3_) in serum were significantly higher by 48.23% and 280%, respectively. The PTH and CRP levels were significantly reduced by 32.5% and 35.27%, respectively, whereas calcitonin was increased by 36.67% compared with the VDD group. Klotho and β-endorphin expressions in cerebrospinal fluid were significantly elevated by 19.67% and 133.59%, respectively, compared to VDD group.

**Conclusions:**

Overall, the results indicate that supplementation of cholecalciferol significantly improved spatial memory impairment, VDR expression, and may provide an opportunity to manage vitamin D deficiencies.

## Highlights

Chronic vitamin D_3_ supplementation significantly improved short-term spatial memory function in VDD rats.

Significant improvement of neuromuscular function and pain score.

VDR expression followed by levels of 25-OH vit.D_3_ were improved.

Klotho and β-endorphin expression were significantly improved.

PTH and CRP levels were significantly reduced.

## Introduction

Currently, vitamin D deficiency is recognized as a global health burden, with high mortality rates related to bone and immune disorders, autoimmune disorders, hypertension, and cancer. In contrast, more than 45% of the population is reported to have low vitamin D levels (less than 20 ng/mL) [1]. Probably low serum vitamin D levels are due to the low vitamin and calcium content of non-supplemented foods. Additionally, sunlight exposure significantly raises the level of circulating vitamin D [2]. The high prevalence of vitamin D deficiency is also influenced by cultural and racial factors. Several cultural factors can influence the amount of sunlight the skin receives, such as clothing, age, sex, and use of sunscreen protectors including racial factor (dark skin pigmentation). Due to the dilution in fat mass, the high prevalence of obesity in the population contributes to greater vitamin D deficiency due to lower circulating levels. Vitamin D deficiency, prevalent in the obese and African Americans, is associated with numerous aging-related diseases, such as hypertension, type 2 diabetes mellitus, and coronary heart diseases [3, 4]. The metabolism of calcium, phosphate, and bone is controlled by vitamin D. Numerous studies reported that the majority of students (94%) believed that bone and skeletal disorders could be caused by vitamin D deficiency. According to literature, deficiency of vitamin D is linked to high incidences of diabetes, autoimmune disorders, cardiovascular disorders, psychological disorders, and cancer [5]. Vitamin D supplementation has therefore been reported to be helpful in various immune and brain-related disorders, as it reduces the behavioral scores of rats suffering from neuropathic pain [6]. Vitamin D deficiency is also associated with significant impairment of spatial learning [7]. Furthermore, vitamin D supplementation can improve cognitive decline and hippocampal synaptic function in aging rats [8]. According to research, vitamin D possesses anti-aging/wellness characteristics. It is associated with a gene (klotho) located in cerebrospinal fluid (CSF) that is responsible for anti-ager expression. Anti-senescence protein plays a direct role in aging *via* multiple mechanisms including calcium homeostasis, the vitamin D receptor (VDR) mechanism, hormonal changes, and bone strength [9].

Based on the existed information, the present study (classic depletion-repletion model) was designed to examine the effects of vitamin D_3_ deficient diet (VDD)-induced abnormalities and supplementation of vitamin D_3_ (cholecalciferol) in male Sprague Dawley rats with respect to spatial memory and cognition, muscular function, pain score, VDR expression, and levels of functional physiological biomarkers.

## Methods

### Animals and VDD model

This study was conducted on adult male Sprague Dawley rats (age 10–12 weeks; weight 250–300 g). The animals were procured commercially from Vivo Bio-Tech, Hyderabad, India, and obtained informed consent from the company to use the animals in this study. The study protocol was in line with animal ethics guidelines for experiment. The experiments were conducted in accordance with the guidelines of the Committee for the Purpose of Control and Supervision of Experiments on Animals (CPCSEA). The test facility (Dabur Research Foundation, India) was registered with CPCSEA (Registration No. 64/PO/br/s/99/CPCSEA) for an experiment involving animals, Ministry of Environment and Forest, Govt. of India and the study was approved by the Institutional Animal Ethical Committee (IAEC), Dabur Research Foundation (DRF), India (IAEC/39/468) on dated 3rd July 2017.

Rats were housed under controlled environmental conditions at 25 °C in a 12 h light/dark cycle and water *ad libitum*. Efforts were made to reduce animal suffering and minimize the number of animals used. Normal control animals were fed with standard normal chow diet (type − 1324, batch/lot no. 110,118//0640) and other treatment group animals were fed with a vitamin D_3_ deficient diet (VDD; type - C 1017, batch/lot no. 481/2017), obtained from Altromin Spezialfutter GmbH & Co., KG, Germany, for three weeks. To check the rat’s model three weeks after VDD administration, 25(OH) vitamin D_3_ was measured in the circulation. Reduced 25(OH) vitamin D_3_ levels by about 50–60% in disease control animals (VDD) compared to normal control animals were considered as VDD-induced animals and were selected for further treatment.

### Groups and drug administration

The 48 adult rats were each randomly assigned to the following six groups (n = 8): normal control with 0.5% CMC, VDD model with VDD and 0.5% CMC, VDD + calcitriol (0.5 µg/kg body weight per oral) treatment, VDD + vit.D_3_ (0.25 mg/kg body weight per oral) treatment, VDD + vit.D_3_ (0.5 mg/kg body weight per oral) treatment, and VDD + vit.D_3_ (1 mg/kg body weight per oral) in the treatment groups. Dosage of vitamin D was chosen as per Bakhtiari-Dovvombaygi et al. 2021 with slight modification [10]. Each treatment groups’ animals were treated orally for eight weeks following the induction of the VDD model. In the 8th week, all the animals were tested for behavioral parameters such as Y-maze task, muscle grip strength, and knee joint pain assessment. After fasting overnight, rats were anesthetized using isoflurane (Aerrane, Baxter Healthcare Corporation, USA) by inhalation (3% induction, then 2% maintenance, with oxygen) at the rate of 1.5 L/min. Approximately 250 µL of blood from each rat was collected from retro-orbital plexus with the help of micro capillary tube and serum samples were used for biomarkers analysis. After bleeding, cerebrospinal fluid (CSF) was collected with a stereotaxic instrument for the estimation of Klotho and β-endorphin by ELISA. All animals were sacrificed by a CO_2_ asphyxiation. Necropsy was performed in a manner that avoided the occurrence of post mortem change in the collected tissues. The organs and tissues were examined in situ before dissecting or collecting tissues. On completion of the gross pathology examination, the livers, kidneys, and adipose tissues were collected and stored in -80 °C for ELISA measurement of 25(OH) D_3_, a metabolite of vitamin D_3_. To estimate VDR expression, half of the liver and adipose tissues were used.

### Y-Maze test

Y-maze testing was used to assess impairment of short-term spatial memory. In the present study, a Y-maze consisting of three arms (35 cm long, 25 cm high, and 10 cm wide) was used with a 120° angle. The arm closest to the experimenter was designated as the start arm, in which rats were placed in each trial. During the first trial (5 min), the novel arm entry was closed and the animals were allowed to explore both open and start arms. Within 30 min of opening the novel arm, animals were allowed to explore all three arms. Exploratory behavior was evaluated for five minutes. In week 8, all the animals were subjected to the Y-Maze test for 5 min. The novelty test includes the estimation of time spent in each arm and number of entries made into each arm. By contrast, spontaneous alternation (i.e., sequential entry into three arms in overlapping triplet sets) includes percentage alternation behavior, was calculated using the following equation: (successive triplet sets/total number of arms entries − 2) X 100 (successive triplet sets : entries into three successive arms).

### Muscle grip strength and knee joint pain score

The muscle grip strength was assessed by lifting the tails of rat, so that they could grasp a rigid bar attached to a digital force gauge. In addition, each animal was gently pulled backward by its tail, and the tension reading of the digital force gauge was measured. Before the animals released the bar, that reading was reported in grams as grip strength. The same experimental procedure was repeated five times successively, and the highest muscle grip strength value from each trial was recorded as the grip strength. A pressure application meter (PAM, Ugo Basile, Cat. No. 38,500) was used to measure knee joint pain. By using the PAM probe, pressure was applied to both knee joints of the animal. As a result of pain, the PAM instrument recorded the animal’s limb withdrawal threshold. The data were expressed as a gram force (i.e., pain threshold).

### Serum biomarker

The serum biomarkers were determined in all the animals in different treatment groups. Blood was collected and used to isolate serum for the estimation of parathyroid (PTH) [CEA866Ra, Clond-Clone], calcitonin [CEA472Ra, Clond-Clone], thyroxine [CSB-E05082r, CUSABIO], and CRP [CSB-E07922r, CUSABIO] using ELISA.

### VDR in liver and adipose tissue

According to the manufacturer’s recommended standard procedure (CUSABIO, USA) [CSB-EL025583RA, CUSABIO], we measured VDR concentrations in liver and adipose tissue homogenates. In brief, about 100 µL of each test sample, including the control and reference standard, were poured into the labeled wells and incubated at 37 °C for an hour. After removing the cover and discarding the liquid, and 100 µL of detection reagent-A working solution was poured into all wells and again incubated at 37 °C for an hour. The plate was then washed three times with 1X washing buffer. Each well was then filled with 100 mL of detection reagent B working solution, sealed, and incubated at 37 °C for 30 min. Following the discarding of the solution, the plate was washed and 90 µL of 3,3’5,5’-Tetramethylbenzidine (TMB) substrate added to each well and incubated at 37 °C for 10 min. After that, 50 µL of stop solution was added to each well, and reading was taken at 450 nm.

### Metabolite (25-OH Vit D_3_) analysis in multiple matrix

A serum, liver, and kidney homogenate were used from all the animals for estimating the 25-OH Vit D_3_ metabolite (CSB-E08098r, CUSABIO) using ELISA, as recommended by the manufacturer.

### Klotho and β-endorphin in CSF

CSF was collected by the standard in-house method using a stereotaxic instrument to measure Klotho [CSB-E14958r, CUSABIO] and β-endorphin [CSB-E08206r, CUSABIO] using ELISA.

### Statistics

Statistical analysis was performed using Sigma-Plot (V11.0). Data are shown as mean ± standard error of the mean. Multiple comparisons were analyzed by one-way analysis of variance (ANOVA) followed by *post-hoc* analysis by Tukey’s/Dunnett’s test and between two groups by Student’s *t*-test. F values *p* < 0.05 were regarded as statistically significant.

## Results

### Induction of VDD model

Except for the normal control, all the animals were fed a vitamin D_3_ deficient diet (VDD) without cholecalciferol and calcium chloride for three weeks. The level of 25(OH) vitamin D_3_ in the circulatory system was measured three weeks after VDD administration. Tukey’s *post hoc* test revealed that the level of 25(OH) vitamin D_3_ was significantly (F_(5,30)_ = 14.796, *p* < 0.001) reduced in all the treatment groups than normal control, were considered as VDD-induced animals and selected for further treatment (data not shown).

### Y-maze task

**Fig. 1:**
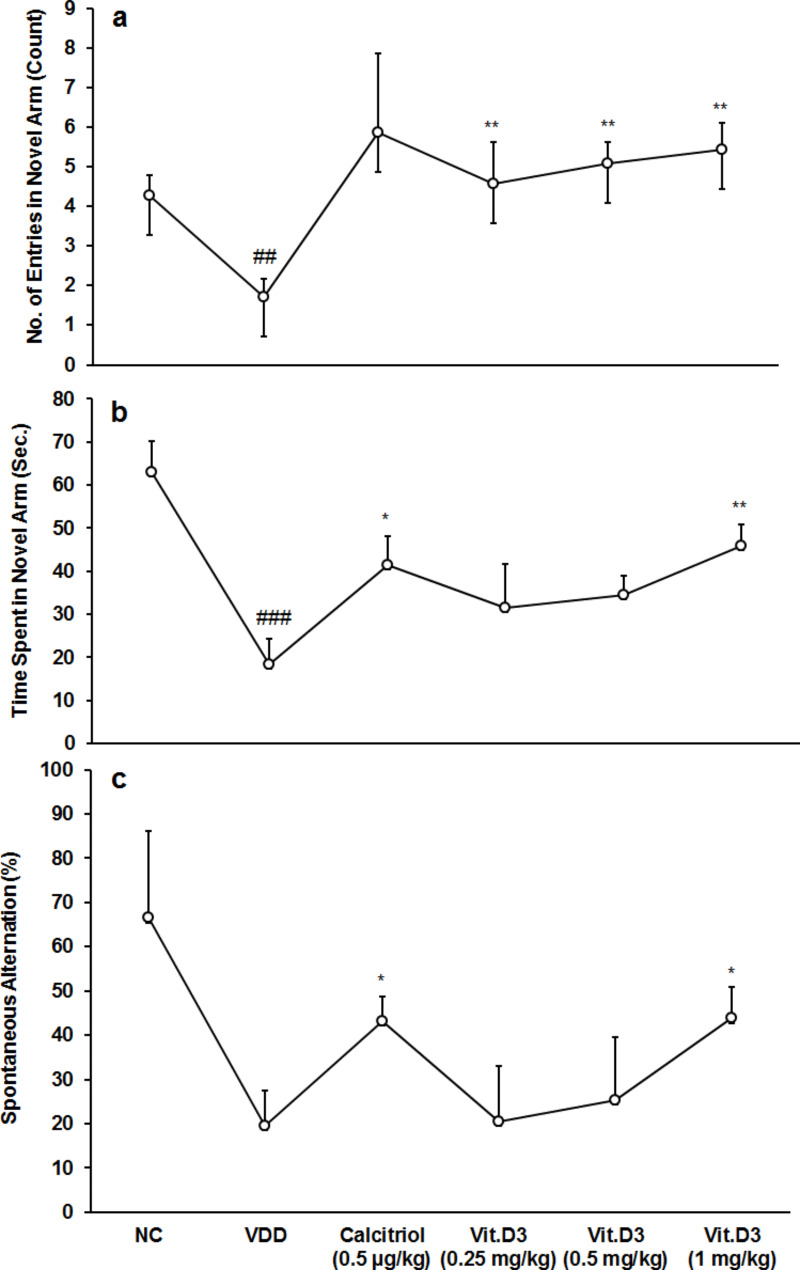
Response to Y-maze task for strain Sprague Dawley rats in relation to **(a)** Number of entry in novel arm, **(b)** Time spent in novel arm, and **(c)** Spontaneous alternation. Data shows mean ± standard error of mean for groups (n = 6). *p ≤ 0.05 and **p ≤ 0.01 vs. disease control (VDD); ^##^*p* < 0.01 and ^###^*p* < 0.001 vs. normal control (NC).

In the novel arm, the number of entries was decreased by 60% in the vitamin D-deprived animals i.e., disease control (1.71 ± 0.44) group than normal control (4.29 ± 0.49). There was a significant difference (CI = 1.103 to 4.040; *t* = 3.901; *p* = 0.003) in the number of entries into the novel arm between the two groups. In the Y-maze task, one-way ANOVA (Tukey’s *post hoc* analysis) revealed a significant (F_(3,20)_ = 5.585; *p* = 0.006) increase of spatial memory performance in vitamin D_3_-treatment groups with low, medium, and high doses by 167.34, 197.11, and 217.46%, respectively than disease control (Fig. 1a), indicating that vitamin D_3_ significantly improved short-term spatial recognition memory.

Time spent in novel arm was decreased by 71.02% in the disease control (18.27 ± 6.16) group than normal control (63.03 ± 7.20). There was a significant difference (CI = 23.651 to 65.883; *t* = 4.724; *p* ≤ 0.001) in time spent into novel arm between two groups. Positive control (41.41 ± 6.66) showed significant (CI = -43.368 to -2.921; *t* = -2.550; *p* = 0.020) increased time spent in novel arm by 126.66% compared to disease control. Pair *t*-test analysis revealed a significant difference between disease control and vit.D_3_ (1 mg/kg) group (CI = -45.358 to -9.808; *t* = -3.458, *p* = 0.006). Time spent in novel arm was increased by 72.47, 89.16, and 150.96% in vitamin D_3_-treatment groups with low, medium, and high doses, respectively than disease control (Fig. 1b).

Continuous spontaneous alternation behavior *-* Working memory capacity is determined by the percentage of spontaneous alternation. The percentage of spontaneous alternation was decreased by 70.56% in the disease control (19.58 ± 7.87) group than normal control (66.49 ± 19.78). Positive control (43.11 ± 5.72) showed significant (CI = -45.196 to -1.864; ***t*** = -2.420; *p* = 0.036) increased spontaneous alternation by 120.15% compared to disease control. Pair *t*-test analysis revealed a significant difference between disease control and vit.D_3_ (1 mg/kg) group (CI = -47.998 to -0.450; *t* = -2.270, *p* = 0.047). Percentage of spontaneous alternation was increased by 29.42 and 123.70% in vitamin D_3_-treatment groups with medium and high doses, respectively, than disease control (Fig. 1c), suggesting significant effects on spatial working memory.

### Assessment of neuromuscular function and knee joint pain

**Fig. 2:**
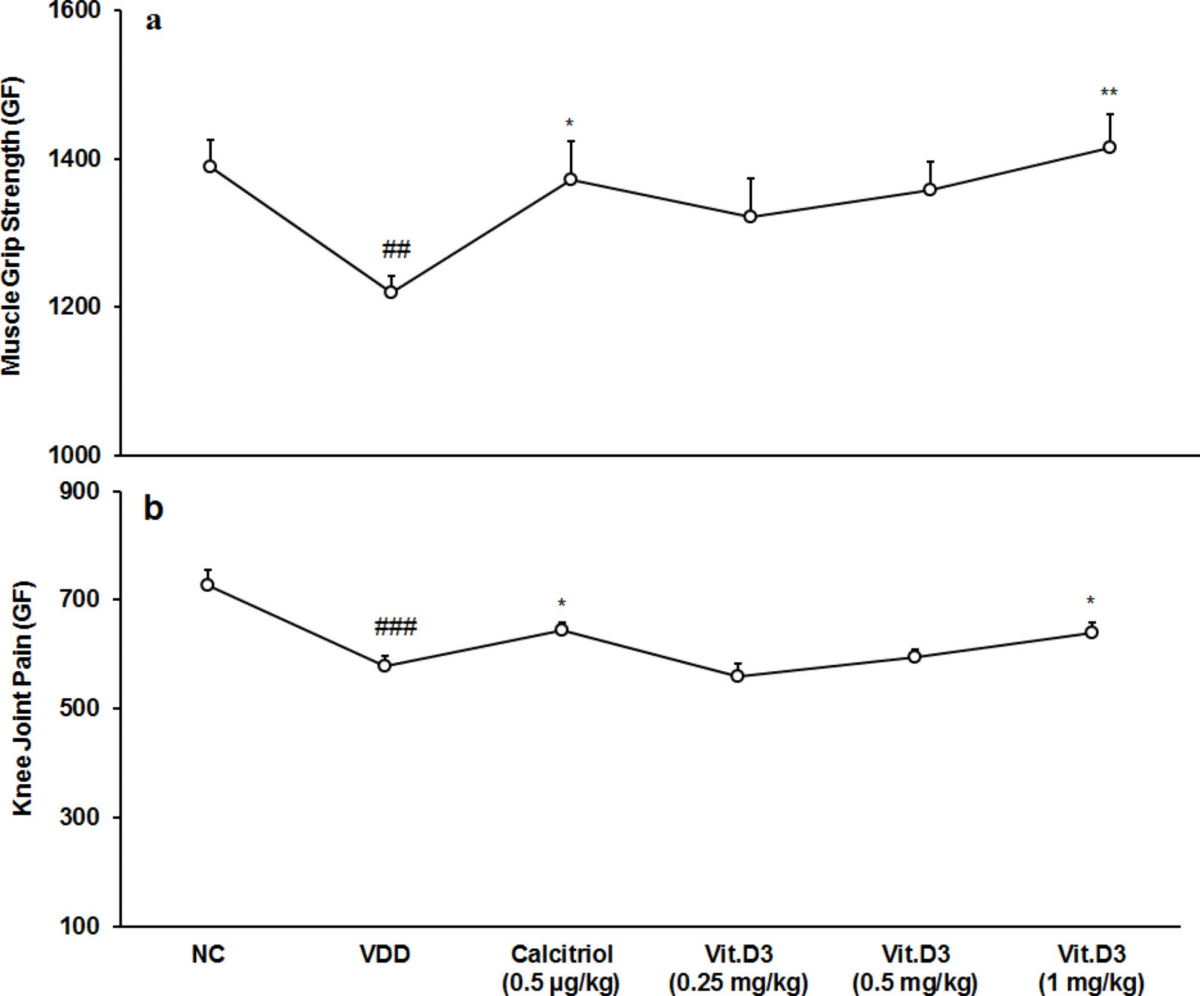
Response to **(a)** Neuromuscular function by muscle grip strength task and **(b)** Knee joint pain score by pressure application meter (PAM) for strains Sprague Dawley rats. GF: Gram force; *p ≤ 0.05 and **p ≤ 0.01 vs. disease control (VDD), ^##^*p* < 0.01 and ^###^*p* < 0.001 vs. normal control (NC).

Neuromuscular function by muscle grip strength task *-* Muscle strength is an essential step for researching neuromuscular disorders. It was measured and reported in terms of gram force (GF). In the results found at the end of the experiment, despite the VDD treated animals (1219.06 ± 22.33) having presented lower grip strength by 12.30% than normal control (1390.06 ± 35.38). There was a significant difference (CI = 77.773 to 264.213; *t* = 4.087, *p* = 0.002) in muscle grip strength between two groups. Positive control (1371.56 ± 52.13) showed significant (CI = -278.861 to -26.139; *t* = -2.689; *p* = 0.023) increased muscle grip strength by 12.51% compared to disease control. Pair *t*-test analysis revealed a significant difference between disease control and vit.D_3_ (1 mg/kg) group (CI = -308.741 to -82.598; *t* = -3.856, *p* = 0.003) (Fig. 2a).

Knee joint pain score by pressure application meter - Knee joint pain in terms of GF was significantly (CI = 74.514 to 222.436; *t* = 4.473, *p* = 0.001) decreased by 20.42% in disease control than normal control. Positive control showed significant (CI = -118.083 to -11.745; *t* = -2.720; *p* = 0.022) increased GF by 11.22% compared to disease control. Pair *t*-test analysis revealed a significant difference between disease control and vit.D_3_ (1 mg/kg) group (CI = -119.973 to -1.747; *t* = -2.294, *p* = 0.045) (Fig. 2b).

### Estimation of VDR and 25-OH vitamin D_3_ in in multiple matrix

**Fig. 3:**
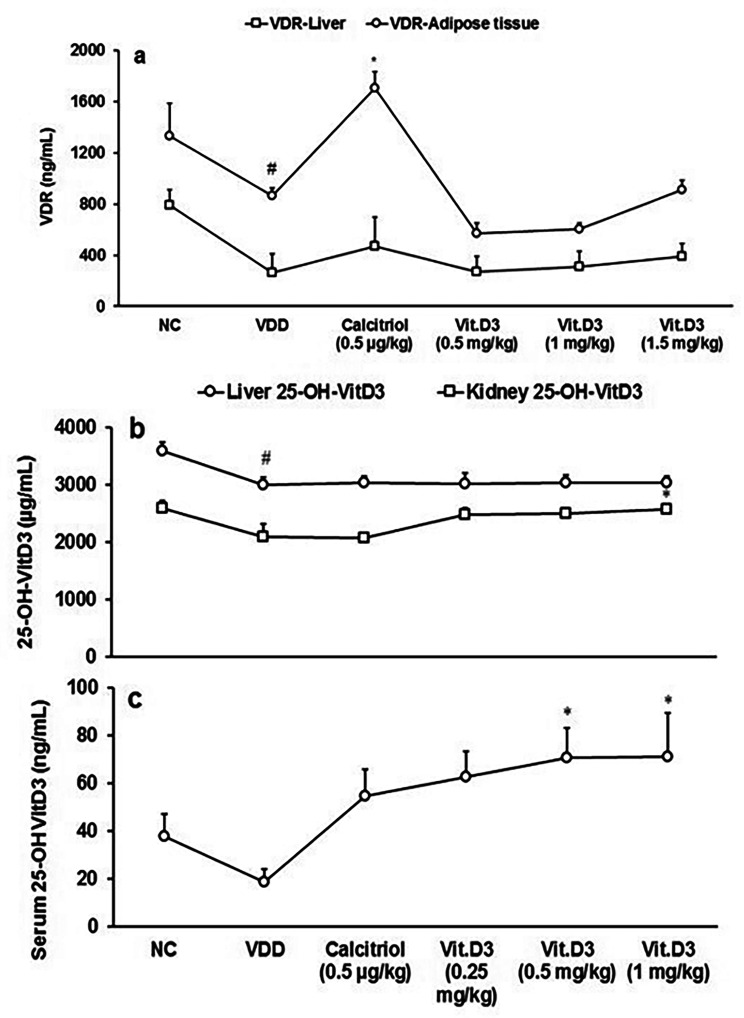
Response to vitamin D_3_ receptor (VDR) concentration in terms of ng/mL and 25-OH vitamin D_3_ levels after 56 days successive oral administration of vitamin D_3_ in vitamin D_3_ deficiency diet (VDD)-induced male Sprague Dawley rats. *p ≤ 0.05 vs. disease control (VDD), ^#^*p* < 0.05 vs. normal control (NC). **(a)** VDR in liver and adipose tissue (ng/mL) **(b)** 25-OH vitamin D_3_ in liver and kidney (µg/mL) and **(c)** 25-OH vitamin D_3_ in serum (ng/mL). *p ≤ 0.05 vs. disease control (VDD) and ^#^*p* ≤ 0.05 vs. normal control (NC).

The results of VDR expression (ng/mL) in the liver and adipose tissues were evaluated and are presented graphically in Fig. 3a. VDR expression in adipose tissue was significantly (CI = 49.241 to 885.739; *t* = 2.490, *p* = 0.032) decreased by 35.09% in the disease control than normal control. Positive control showed significant (CI = -1449.629 to -228.171; *t* = -3.061; *p* = 0.012) increased VDR expression by 97% compared to disease control. VDR expression in liver was increased by 48.23% in vitamin D_3_-treatment at 1 mg/kg than disease control (Fig. 3a).

Level of 25-OH vitamin D_3_ in liver tissue was significantly (CI = 121.045 to 1052.955; *t* = 2.807, *p* = 0.019) decreased by 16.40% in the disease control group than normal control. Dunnett’s *post hoc* test revealed that the level of 25-OH vitamin D_3_ in kidney tissue was significantly (F_(4,25)_ = 3.566, *p* = 0.020) increased by 23.24% in the vit.D_3_ (1 mg/kg) group compared to VDD (Fig. 3b). Besides, Tukey’s *post hoc* test revealed that the level of 25-OH vitamin D_3_ in serum was significantly (F_(4,25)_ = 3.104, *p* = 0.033) increased by 278.12% and 281.11% in the 0.5 and 1 mg/kg, respectively compared to VDD (Fig. 3c).

### Estimation of serum biomarkers

**Fig. 4:**
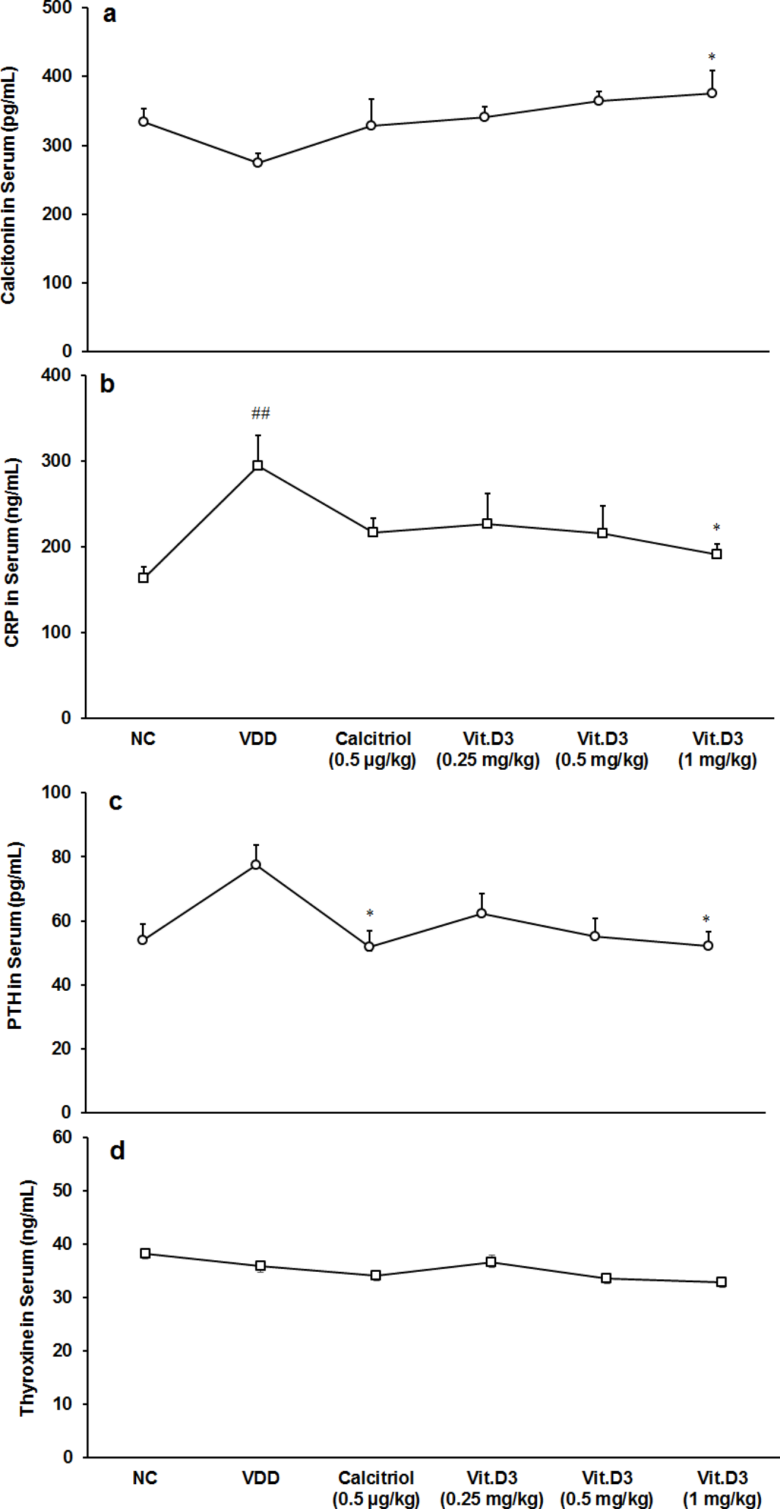
Response to serum stress-related biomarkers in Sprague Dawley rats (n = 6). **(a)** Calcitonin (pg/mL); **(b)** C-reactive protein (CRP, ng/mL); **(c)** Parathyroid hormone (PTH, pg/mL); and **(d)** Thyroxine (ng/mL). ^##^*p* ≤ 0.01 vs. normal control (NC) and *p ≤ 0.05 vs. disease control (VDD).

The results of serum biomarkers are graphically presented in Fig. 4. Levels of calcitonin and parathyroid hormone (PTH) were expressed in terms of pg/mL. C-reactive protein (CRP) and thyroxine were expressed in terms of ng/mL. Level of CRP was significantly (CI = -215.856 to -47.804; *t* = -3.496, *p* = 0.006) increased by 81.02% in the disease control than normal control.

Tukey’s *post hoc* test revealed that the level of calcitonin was significantly (F_(1,10)_ = 8.011, *p* = 0.018) increased by 36.66%, while CRP was significantly (F_(1,10)_ = 7.825, *p* = 0.019) decreased by 35.27% at 1 mg/kg than disease control (Fig. 4a, b). Further, Tukey’s *post hoc* test revealed that the level of PTH was significantly (F_(4,25)_ = 3.732, *p* = 0.016) decreased by 33.11% and 32.50% in the positive control and vitamin D_3_ (1 mg/kg) groups, respectively than disease control (Fig. 4c).

### Estimation of klotho and β-endorphin in CSF

**Fig. 5:**
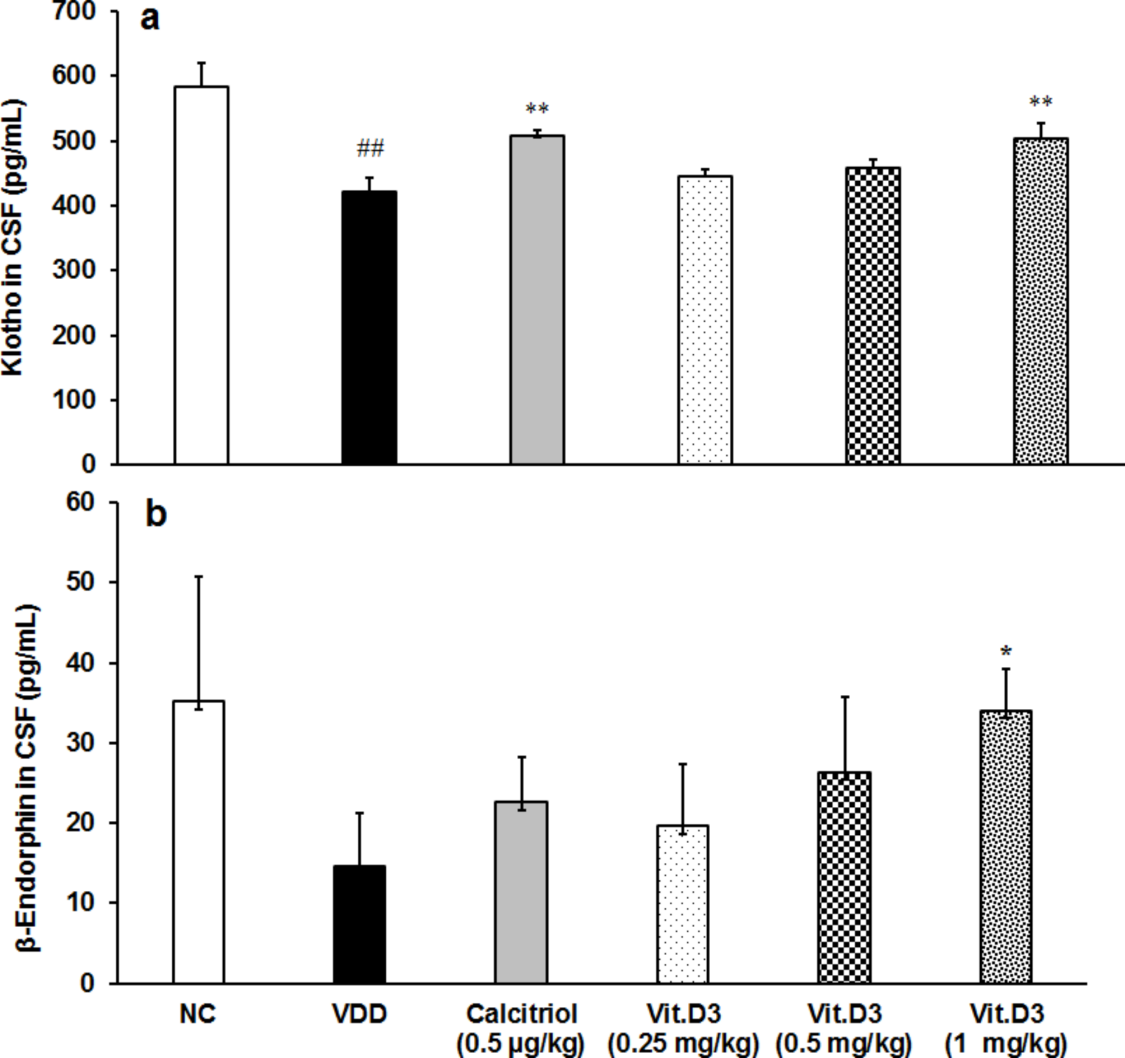
Response to **(a)** Klotho protein and **(b)** β-Endorphin levels in cerebro spinal fluids (CSF) in Sprague Dawley rats (n = 6). *p ≤ 0.05 and **p ≤ 0.01 vs. disease control (VDD) and ^*##*^*p ≤ 0.01 vs. normal control (NC).*

The results of Klotho and β-endorphin in CSF were evaluated and are presented graphically in Fig. 5. The expression of both the biomarkers was calculated in terms of pg/mL. Expression of klotho protein in CSF was significantly (CI = 68.066 to 256.734; *t* = 3836, *p* = 0.003) decreased by 27.80% in the disease control than normal control. Positive control showed significant (CI = -138.379 to -33.521; *t* = -3.653; *p* = 0.004) increased klotho protein by 20.37% compared to disease control (Fig. 5a).

The expression of klotho (Tukey’s post hoc test) and β-endorphin (pair *t*-test) showed significantly increased by 19.67% (F_(4,5)_ = 5.247, *p* = 0.003) and 133.59% (CI = -38.119 to -0.859; *t* = -2.331, *p* = 0.042), respectively at 1 mg/kg than disease control (Fig. 5a, b).

## Discussion

**Fig. 6:**
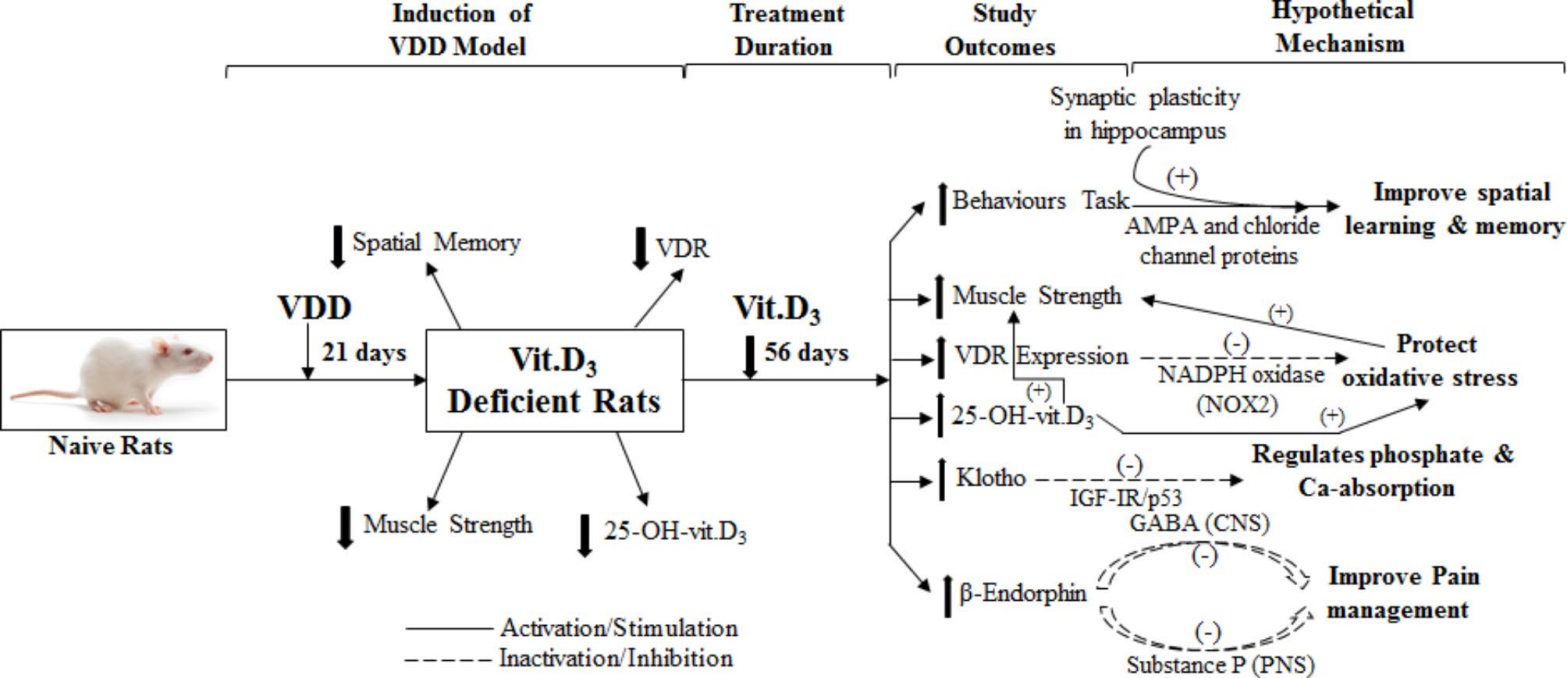
*Schematic representation of experimental design, study outcomes, and possible mechanistic pathways*

Recently, there have been growing concerns about vitamin D deficiency as a risk factor associated with cognitive impairment/aging. A few studies have investigated the effects of successive vitamin D supplements (vit.D_3_) on modifying diet regimens in deprived cholecalciferol rats. Therefore, it is unclear whether a potential relationship presents between depletion and supplementation of vitamin D_3_. We tested the hypothesis that cognitive decline associated with low cholecalciferol can be improved by long term (56 day) administration of cholecalciferol. In addition to, cognitive memories, authors also measured pain score, muscle function, hormonal changes, VDR expression, metabolite levels, and anti-aging protein. Briefly, induction of VDD model, treatment duration, study outcomes, and probable mechanisms are shown schematically in Fig. 6.

The Y-maze is a standard test for determining spatial working and reference memory [11]. The Y-maze spontaneous alternation is a behavioral test that measures the willingness of rodents to explore new environments to develop spatial working memory. The novelty test was used to determine the time spent and the number of entries in each arm [12]. In the present study, we demonstrate that the depletion of foodstuff regimen with vitamin D and calcium results in pronounced reduced spatial performance. The VDD animals spent less time hunting the novel target and made fewer entries. However, vitamin D supplementation significantly influences the maze performance in learning the maze task. Growing evidence that supports the positive effects of vitamin D on learning and behavior. In this study, the improvement in performance on the Y-maze task could be attributed to the involvement of vitamin D receptors and catalytic enzymes in the brain (hippocampus) responsible for complex planning, processing, and memory formation [7]. Researchers have found that spatial memory function can be improved by upregulating the expression of AMPA and chloride channel proteins in the hippocampus [13].

Coradini et al. (2015) reported weak muscle strength in nerve compression model due to hypernociception [14]. Vitamin D is directly involved in muscle function. Weak muscles or altered muscle fibers due to vitamin D deficiency or ablation of vitamin D receptors (VDR) [15]. Furthermore, supplementing with vitamin D helps to counteract fatigue and weakness [16]. Compared with normal animals, VDD-treated animals had decreased grip strength due to a lack of vitamin D. Consequently, muscle grip strength was significantly increased by vitamin D_3_ supplementation in a dose-dependent manner. The knee joint pain measurement by pressure application meter (PAM) is a novel behavioral rat model to evaluate joint pain hypersensitivity. The reading of PAM as gram force (GF) is considered the gold standard for behavioral hypersensitivity. In addition, the method is a highly reproducible and a useful tool for chronic inflammatory joint pain evaluation [17]. In literature, pain pathways have been described as various mechanisms including pain-sensing and processing primarily at the level of dorsal root ganglion neurons. On the other hand, authors have reported the significant role of vitamin D and its receptor (VDR) in specific pain signaling pathways that include epidermal growth factor receptor (EGFR), glial-derived neurotrophic factor (GDNF), nerve growth factor (NGF), and opioid receptors [18]. Thus, vitamin D_3_ treatment regimen is highly effective in pain-reducing activity *via* VDR receptor.

It was reported in many studies that VDR regulates the expression of numerous genes that are responsible for cellular proliferation, calcium/phosphate homeostasis, and immune response [19]. The biological effects of 25-hydroxy vitamin D_3_ and 1,25 dihydroxy vitamin D_3_ are mainly mediated by the VDR. Thus, chronic repeated oral administration of vitamin D_3_ significantly improved the level of VDR expression in the liver and adipose tissues that mediate various biological functions. As a result of vitamin D_3_ supplementation behavioral activity, VDR level, and joint pain can also be improved. Literature data reported that vitamin D has a significant therapeutic role in many neurological diseases *via* inhibition of the NADPH oxidase (NOX2), which protects against reactive oxygen species (ROS) production [20, 21]. Hence, the assumption is that vitamin D_3_ supplementation reduces animal activity through VDR mechanisms.

The secretion of calcitonin (antagonist of PTH) is stimulated by an elevated calcium level and low blood calcium. It is indirectly involved in the regulation of bone mineralization and synthesis of bone matrix [22, 23]. VDD group animals showed reduced levels of calcitonin, while supplementation with vitamin D_3_ significantly increased the level of calcitonin compared with the VDD group. However, CRP is a pentameric protein found in blood plasma, and its level depends on the body’s inflammation. It is defined as an acute phase reactant and depends on the inflammation level [24, 25]. In this study, the acute-phase reactant CRP was significantly increased after treatment with VDD, where it was significantly reduced following vitamin D_3_ treatment at 1 mg/kg.

PTH, a type of peptide hormone is synthesized from parathyroid gland and regulates the plasma calcium [26]. However, intact PTH (iPTH) is defined as an active form, which is released in blood in case of a low level of calcium. PTH regulates the formation of osteoclasts that enhance bone resorption. Dysregulation of PTH results in hyper/or hypothyroidism [27, 28]. Our results revealed a significant reduction of PTH in vitamin D_3_ treatment groups, which was abruptly increased in the VDD-treated group. Thyroid hormones play an imperative role in muscular activity, metabolic rate, brain development, and maintenance of bone health [29, 30]. The level of thyroxine was slightly changed after vitamin D_3_ treatment with respect to the VDD.

Vitamin D deficiency is defined when the serum levels of 25-OH vitamin D_3_ are less than 20 ng/mL (50 nmol/L). Vitamin D_3_ is metabolized to form 25-OH vitamin D_3_ (liver) and 1,25-dihydroxyvitamin D_3_ (kidney) [31–33]. Insufficient levels of 25(OH) vit.D_3_ increased oxidative/nitrosative stress and inflammation. Supplemental with vitamin D_3_ decreased oxidative DNA damage [34]. Growing literature from randomized controlled trials stated that an increased plasma levels of 25(OH) vit.D_3_ improves muscle strength and or physical performance [35]. Our experimental results suggested that chronic supplementation with vitamin D_3_ significantly improved the metabolite level in serum, liver, and kidney tissues compared with the VDD group. β-endorphin is an endogenous opioid neuropeptide hormone produced in certain neurons within the peripheral nervous system (PNS) and central nervous system (CNS) to relieve pain. In PNS, β-endorphin produce an analgesic effect by binding with opioid (µ) receptor. After binding, a cascade of interactions occurs that inhibits the release of a key protein for pain transmission i.e., substance P. In CNS, β-endorphin similarly bind to the µ-opioid receptor and exert their analgesic effect through inhibits the release of γ-aminobutyric acid (GABA), resulting in excess production of dopamine, which is associated with pleasure [36]. Emerging evidence addresses the Klotho (KL) gene, known as an “aging suppressor” gene that accelerates aging when disrupted and extends lifespan when expressed overly [37]. The level of Klotho was measured in CSF and found to be significantly increased, suggesting the role of vitamin D and VDR. The study suggested that VDR has a significant role in controlling the *KL* gene expression in mouse and human renal cells [38]. Clinical trial data also suggested that vitamin D supplementation significantly affects the level of the brain klotho protein [39]. Several evidences also suggest that α-klotho inhibits insulin/insulin-like growth factor-1 (IGF-1) signaling [40], and oxidative stress [41], and regulates phosphatase and calcium absorption [42, 43]. Thus, overexpression of klotho downregulates the IGF-1R, lowers p53, and reverses the senescence phenotype [44]. Our findings suggested that supplementation with vitamin D_3_ significantly improved the levels of VDR expression, in addition to the β-endorphin, klotho (an anti-aging gene).

### Strength and limitation

The current study was significantly improved memory function, muscle function, pain score. It also improved VDR expression followed by levels of 25-OH vit.D_3_ and other functional physiological biomarkers. While vitamin D and calcium deficiencies, which we assumed as a single factor, contribute to the effects observed in the VDD group, they are due to multiple factors. Information on the vitamin D content in the maintenance diet fed to the control group is not standardized. From a mechanistic perspective, further study like estimation of biomarkers in skeletal muscle and hippocampus is needed to clear such a paradox.

## Conclusions

The findings of this study demonstrate that supplementation with cholecalciferol (vit.D_3_) alleviates neurobehavioral deficits and brain cognition in vitamin D_3_ deficient diet (VDD)-induced Sprague Dawley rats. It also significantly increases expression of VDR, β-endorphin, and anti-aging protein (Klotho). In light of this, cholecalciferol supplementation may be promising for treating cognitive impairment like Alzheimer’s disease and vitamin D deficiency disorders. Overall, this study shows a strong association between vitamin D_3_ status and the incidence of spatial memory and cognition abilities as well as muscle strength and chronic joint pain in rats.

## Data Availability

The datasets used and/or analyzed during the current study are available from the corresponding author on reasonable request.

## Abbreviations

ANOVA: Analysis of variance
CSF: Cerebrospinal fluid
CRP: C-reactive protein
CI: Confidence interval
CMC: Carboxy methyl cellulose
ELISA: Enzyme-linked immune sorbent assay
EGFR: Epidermal growth factor receptor
GF: Gram force
GDNF: Glial-derived neurotrophic factor
IGF-1: Insulin-like growth factor-1
NGF: Nerve growth factor
NOX2: NADPH oxidase
PAM: Pressure application meter
PTH: Parathyroid hormone
ROS: Reactive oxygen species
VDD: Vitamin D deficient diet
VDR: Vitamin D receptor
